# Limb salvage and complication management after (sub-) total humerus resection for primary malignant bone tumors in early childhood

**DOI:** 10.1186/s13018-025-05956-0

**Published:** 2025-05-28

**Authors:** Wiebke K. Guder, Nina M. Engel, Jendrik Hardes, Lars E. Podleska, Dimosthenis Andreou, Markus Nottrott, Arne Streitbürger

**Affiliations:** Department of Orthopedic Oncology, University Hospital Essen, Hufelandstrasse 55, 45147 Essen, Germany

**Keywords:** Childhood, Complication management, Ewing sarcoma, Limb salvage, Osteosarcoma, Primary bone tumor, Total humerus resection

## Abstract

Limb-salvage after (sub-) total humerus resection in skeletally immature patients, aged less than 10 years, remains the domain of individual case decisions due to a small number of affected patients. Seven children aged *≤* 10 years at the time of (sub-) total bone sarcoma resection of the humerus between 2018 and 2023 were included in this retrospective study. Limb salvage with an intact motor function of the lower arm was achieved in 100% of cases at a mean follow-up of 89.8 months. Reconstruction survival was 28.6%. 61.8% (*n* = 8/13) of complications necessitated revision operations (*n* = 7). Distal instability and dislocation (*n* = 5) was the most frequently observed complication, followed by periprosthetic infection (*n* = 2). However, both fixed hinge prostheses and hemiprostheses with a highly cancellous implant body led to stable reconstructions of the elbow joint. Arm lengthening (5 cm) was successful in two patients who underwent implantation of a growing prosthesis. Patients developed a handedness contralateral to the operated arm in this study. The mean MSTS and TESS scores were 16.6 and 51.5. Patients suffered from a limited range of motion in the glenohumeral joint, limited fine motor function and strength despite an intact innervation of the executing muscles. Salvage of even short segments of the distal humerus has a high potential to decrease complication rates, whenever oncologically feasible. The use of fixed-hinge total humerus (growing) prostheses and custom-made total humerus hemiprostheses with a highly cancellous implant body showed the most promising results in terms of complications and functional outcomes. Arm lengthening using growing prostheses - albeit of a mostly cosmetic value - is an option in the upper extremity whenever the soft tissue coverage of the implant is adequate.

## Background

Limb-salvage after (sub-) total humerus resection in skeletally immature patients, aged less than 10 years, remains the domain of individual case decisions.

In part, this is due to a small number of affected patients and limited reports on their reconstruction outcomes in literature. While the humerus is the second most common tumor site in high-grade osteosarcoma with an incidence of 15%, it is particularly uncommon in patients aged < 10 years [1]. Moreover, the incidence of the humerus as the primary tumor site in Ewing sarcoma is equally low with an overall incidence of 5.8% and 7.6% in patients aged 0–9 years [2].

While the greatest challenges in (sub-) total humerus resection in a pediatric patient cohort are the smaller dimensions and proportions of bone, soft tissues and neurovascular structures, the aim of limb salvage is similar to that of adolescent and adult collectives. The main goals are to maintain arm length and thusly preload for flexion and extension of the elbow joint [3], as well as an intact motor function of the lower arm, hand and fingers. Partial resection and detachment of the muscles of the rotator cuff - and especially loss of the axillary nerve - usually lead to a very limited range of motion in the glenohumeral joint. Randall even proposes that the functional outcome a patient might achieve after surgery seems to be more dependent upon the extent of the resection than on the particular reconstruction itself [4].

In literature, both biological and endoprosthetic reconstruction techniques of the proximal (and rarely total) humerus have been described. These include fixation of the distal humerus to the second rib or clavicle with Küntscher nails [3], as well as a clavicula pro humero (CPH) reconstruction alone or in combination with a vascularized fibula autograft [3, 5–8]. The main advantages and disadvantages reported are a reliable, permanent reconstruction once union is achieved versus a shortening of the upper limb and varying periods until bone union is achieved. Among complications, non-union, clavicle fracture and/or osteolysis and aseptic pseudarthrosis are listed [5–8]. Other biological reconstructions include (composite) allografts and free or vascularized fibula grafts/vascularized fibular epiphyseal transfer (VFET) [9–10]. Fractures, avascular necrosis and slippage of the fibular epiphysis of the autograft as well as a donor-site morbidity (lateral tibial physis growth arrest) are reported among complications for this reconstruction type, while growth of the reconstruction after VFET and permanence of reconstruction are listed as their main benefits [9]. Another reported reconstruction type are expandable and (total) humerus endoprostheses [11–17]. Among advantages, an early return to function and reduction of limb length discrepancy for expandable prostheses are reported. Periprosthetic infection, fracture, material wear and migration are the main complications [11–17].

In an effort to improve evidence-based decision making and counseling in this unique patient cohort, it is the objective of this study, to present the outcomes of both novel and existing reconstruction designs chosen for primary and revision operations. We report the incidence of reconstruction-specific complications and their management, as well as the functional outcomes observed. By doing so, we provide novel and important information about the success rates of key design aspects of custom-made implants in the total humerus and distal humerus/elbow joint. This data may serve as a guide to fellow orthopedic oncologists and supports the continued development of innovative, well-performing reconstruction techniques in challenging situations.

## Materials and methods

Seven children aged *≤* 10 years at the time of primary bone sarcoma resection of the humerus who underwent primary tumor resection and/or revision surgery at this tertiary hospital between 2018 and 2023 were identified from a surgical database. We performed a retrospective analysis of patient data, which was collected prospectively in the hospital information system.

### Inclusion criteria

Inclusion criteria in this analysis were.

1. presence of a primary bone sarcoma of the humerus.
2. patient age *≤* 10 years at the time of primary tumor resection.

All patients, who met these inclusion criteria and received either primary or revision surgery between 2018 and 2023 were included. We did not define exclusion criteria within this group of patients.

### Patient characteristics

Mean patient age at the time of primary tumor resection in this cohort was 5.4 years (range 3–10 years). The male: female ratio was 4:3. The patients did not suffer from any pre-existing comorbidities. Ewing sarcoma was confirmed in four and osteosarcoma in three cases by incisional biopsy. All patients completed neoadjuvant and adjuvant chemotherapy protocols for their respective diagnosis. Total humerus resections were performed in five and subtotal proximal humerus resections in two patients. The mean resection length was 197.3 mm (range 125–255 mm). Primary reconstruction was performed using a polymethyl methacrylate (PMMA) spacer in four, a custom-made 3D printed EPORE^®^ total humerus implant in two and a custom-made 3D printed polished total humerus implant in one patient(s). 85.7% of patients (*n* = 6/7) were reconstructed using a megaendoprosthetic custom-made implant at least once during the course of their treatment. At the time of their last follow-up, 71.4% of patients (*n* = 5) were reconstructed using a megaendoprosthesis. Complete resections of the tumor were achieved in all patients. However, histopathological analysis detected hemangiosis sarcomatosa, an infiltration or tumor growth alongside of tumor-feeding vessels, in two patients. Microscopic vascular tumor spread (V1 status) was diagnosed in patient #7, whereas the presence of a macroscopic tumor thrombus in the axillary vein (V2 status) was observed in patient #6. Response to chemotherapy according to Salzer Kuntschik [18] was good in five and poor in two patients. Adjuvant radiation therapy of the tumor bed was performed in both patients with a poor chemotherapy response of their Ewing sarcoma and both patients with a positive V status (one osteosarcoma and Ewing sarcoma each). One patient was diagnosed with synchronous pulmonary metastases, whereas two patients developed metachronous pulmonary metastases during follow-up. One patient (#6, osteosarcoma, V2) was diagnosed with a local recurrence and recurrent pulmonary metastases 25 months after primary tumor resection and 5 months after resection of oligometastatic pulmonary metastases (20 months after resection of the primary tumor) (see Tables 1 and 2).

**Table 1:** Patient and treatment-associated characteristics

#	Age (ys)	Gender	Site	Handedness	Diagnosis	Margin	Response (Salzer Kuntschik)	Radiation	Metastasis	FU (mo)	Age at last FU (ys)
**1**	5	female	left	ambi	Osteosarcoma	R0	2 (3%)	-	-	NED 123	16
**2**	3	male	right	left	Ewing	R0	4 (10–15%)	Y	-	NED 111	12
**3**	4	male	left	right	Ewing	R0	1 (0%)	-	-	NED 101	12
**4**	4	female	left	right	Ewing	R0	4 (20–25%)	Y	-	NED 87	11
**5**	10	female	-	-	Osteosarcoma	R0	3 (5%)	-	pulm meta	DOD 34	-
**6**	7	male	right	left	Osteosarcoma	R0 V2	3 (5%)	Y	pulm meta	AWD 27	9
**7**	5	male	-	-	Ewing	R0 V1	3 (5%)	Y	pulm synch	DOD 14	-

Abbreviations: #: number; ys: years; FU: follow-up; mo: months; ambi: ambidextrous; NED: no evidence of disease; Y: yes; pulm: pulmonary; meta: metachronous; snych: synchronous; DOD: dead of disease

**Table 2:** Reconstruction, complication management and functional outcome overview

#	Age (ys)	# of operations	Resection Length (mm)	1st reconstruction	2nd operation (mo)	3rd operation (mo)	4th operation (mo)	5th operation (mo)	Complications	MSTS	TESS
**1**	5	2	subtotal (125)	*PMMA spacer	Xpand^®^ + CM plated hollow stem (61)	-	-	-	1st distal rod migration 2nd proximal instability + dislocation	26	84
**2**	3	2	subtotal (150)	*PMMA spacer	single stage revision (56)	-	-	-	1st central rod fracture 1st implant-associated infection	14	66
**3**	4	5	total (184)	*PMMA spacer	*polished total humerus (14)	*PMMA spacer (1)	Closed reduction (7;6)	Xpand^®^ total humerus (14;7)	1st, 2nd and 3rd distal instability + dislocation 2nd periprosthetic infection 5th ASL ulna stem	19	67
**4**	4	3	total (190)	*polished total humerus	*PMMA spacer (12)	CPH (16)	-	-	1st and 2nd distal instability + dislocation 3rd CPH fracture	7	17.5
**5**	10	2	total (205)	PMMA spacer	Xpand^®^ total humerus (22)	-	-	-	-	-	-
**6**	7	1	total (255)	EPORE^®^ total humerus	-	-	-	-	Local recurrence	17	23
**7**	5	1	total (202)	EPORE^®^ total humerus	-	-	-	-	-	-	-

Abbreviations: #: number; mo: months; MSTS: musculoskeletal tumor society score; TESS: Toronto Extremity Salvage score; PMMA: polymethyl methacrylate; Xpand^®^: growing prosthesis (implantcast GmbH, Buxtehude, Germany); CM: custom-made; CPH: clavicula pro humero reconstruction; EPORE^®^: implant with a highly cancellous titanium alloy surface; *operations performed at a different tertiary hospital

### Surgical indication, technique, Pre-operative planning

**Fig. 1:**
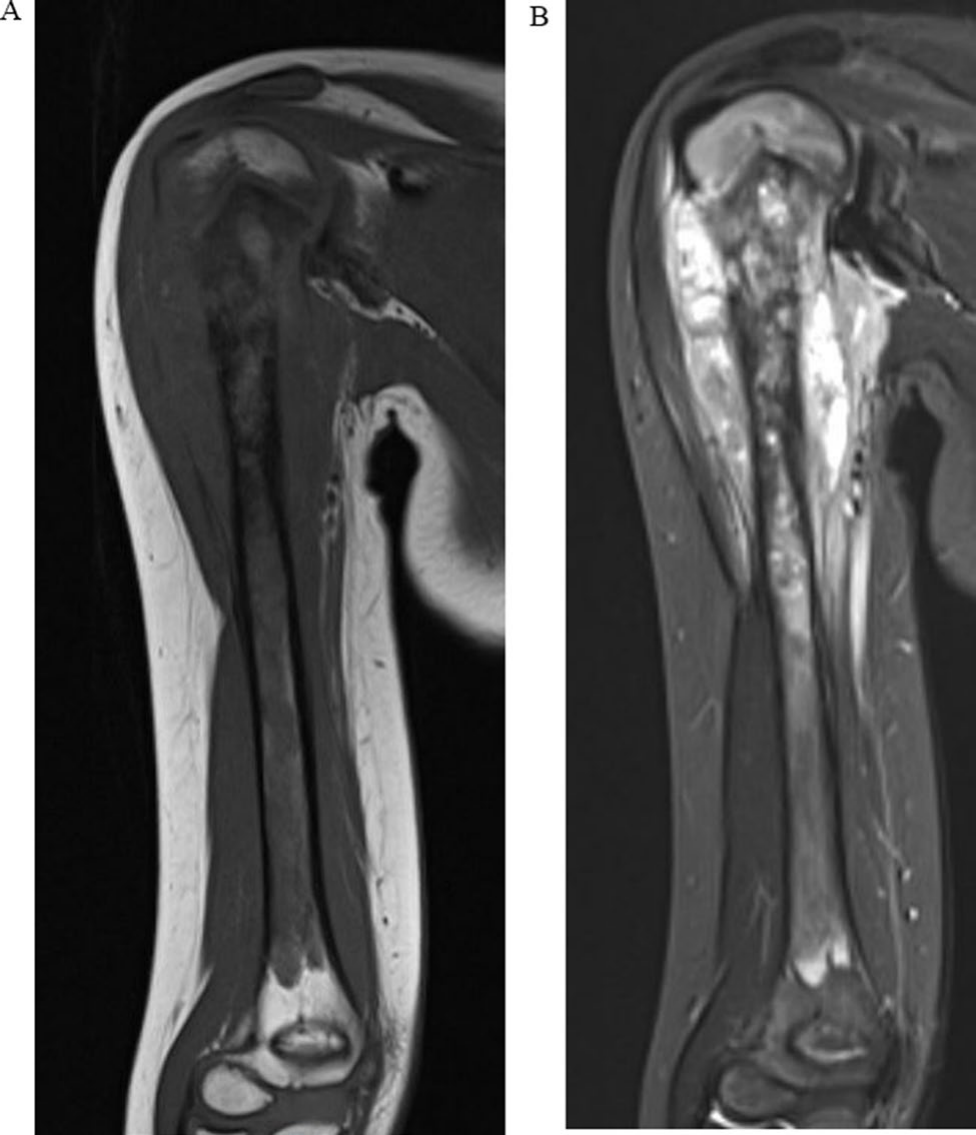
Preoperative tumor extension of a high-grade osteosarcoma of the right humerus (patient #6; MRI: (**a**) coronar T1 TSE, (**b**) coronar TIRM)

(Sub-) total humerus resection in this patient collective was indicated due to locally advanced primary bone sarcomas of the humerus, with large intra- and/or extramedullary tumor components extending to the proximal and/or distal aspects of the humerus (Fig. 1). Tumor extension necessitated total humerus resection in five cases (#3–7). In two cases, subtotal humerus resections salvaging a few centimeters of the distal humerus were feasible while still obtaining an adequate bone safety margin from the most distal bone tumor extension (#1–2).

Reconstruction using a PMMA spacer was the preferred primary reconstruction in the absence of adequately sized endoprosthetic options and late presentation in the outpatient clinic, leading to the unavailability of a custom-made implant at the time of local therapy (according to protocol). Due to a mean resection length of 197.3 mm, biological reconstructions would have led to a significant ad-hoc shortening of arm length, even at the time of primary reconstruction. In addition, information on the necessity of adjuvant radiation therapy in Ewing sarcoma was not available prior to tumor resection and its histopathological analysis. Therefore, primary reconstruction using a PMMA spacer permitted an at least temporary maintenance of arm length and postponement of the decision, whether the PMMA spacer would remain as a permanent reconstruction or patients would undergo a secondary procedure to receive a definite reconstruction/pursue arm lengthening.

Primary or secondary custom-made defect reconstructions using megaendoprosthetic implants were planned on preoperative computed tomography (CT) scans (DICOM format, reconstruction matrix 512 × 512, slice thickness *≤* 1 mm), often using the mirrored, healthy contralateral bone as a template. 3-CAD of the implants was performed by implantcast Inc. (Buxtehude, Germany) as specified and ultimately approved by the attending surgeon. The period for both planning and production of the implant was usually 8–10 weeks. This necessitated the initiation of planning the reconstruction at the time of first diagnosis.

Patients were operated on in a sideways position. An intraarticular resection of the glenohumeral and elbow joint were possible in all cases. While the brachial vessels, musculocutaneus, medianus, radialis and ulnaris nerve were spared in all cases, the axillary nerve was partially (#5–7) or completely (#1–4) sacrificed in three and four cases respectively.

### Postoperative rehabilitation

After PMMA or hemiprosthetic reconstruction of the total humerus, the patients’ elbow joints were immobilized using a 60° plaster splint for four weeks after primary operation and 4–6 weeks plaster cast after an instability had occurred. Afterwards, patients received physiotherapy to exercise extension/flexion of the elbow joint for two weeks, before pronation and supination were included in the physiotherapy regimen. After fixed-hinge reconstruction or salvage of the elbow joint, we did not limit the postoperative range of motion (ROM) and only advised against bearing loads (exceeding e.g. a glass of water) for 6 weeks after the operation.

All reconstructions received the same physiotherapy recommendations with regard to their glenohumerual joint: assisted oscillating movements for two weeks postoperatively, followed by passive-assistive physiotherapy of the shoulder joint omitting elevation (*≤* 90°) during weeks 3–4 and including elevation (> 90°) during weeks 5–6. Starting from week 7, active ROM was approved.

### Reconstruction Types

#### Hemiprosthetic polymethyl methacrylate (PMMA) spacer (*n* = 5)

**Fig. 2:**
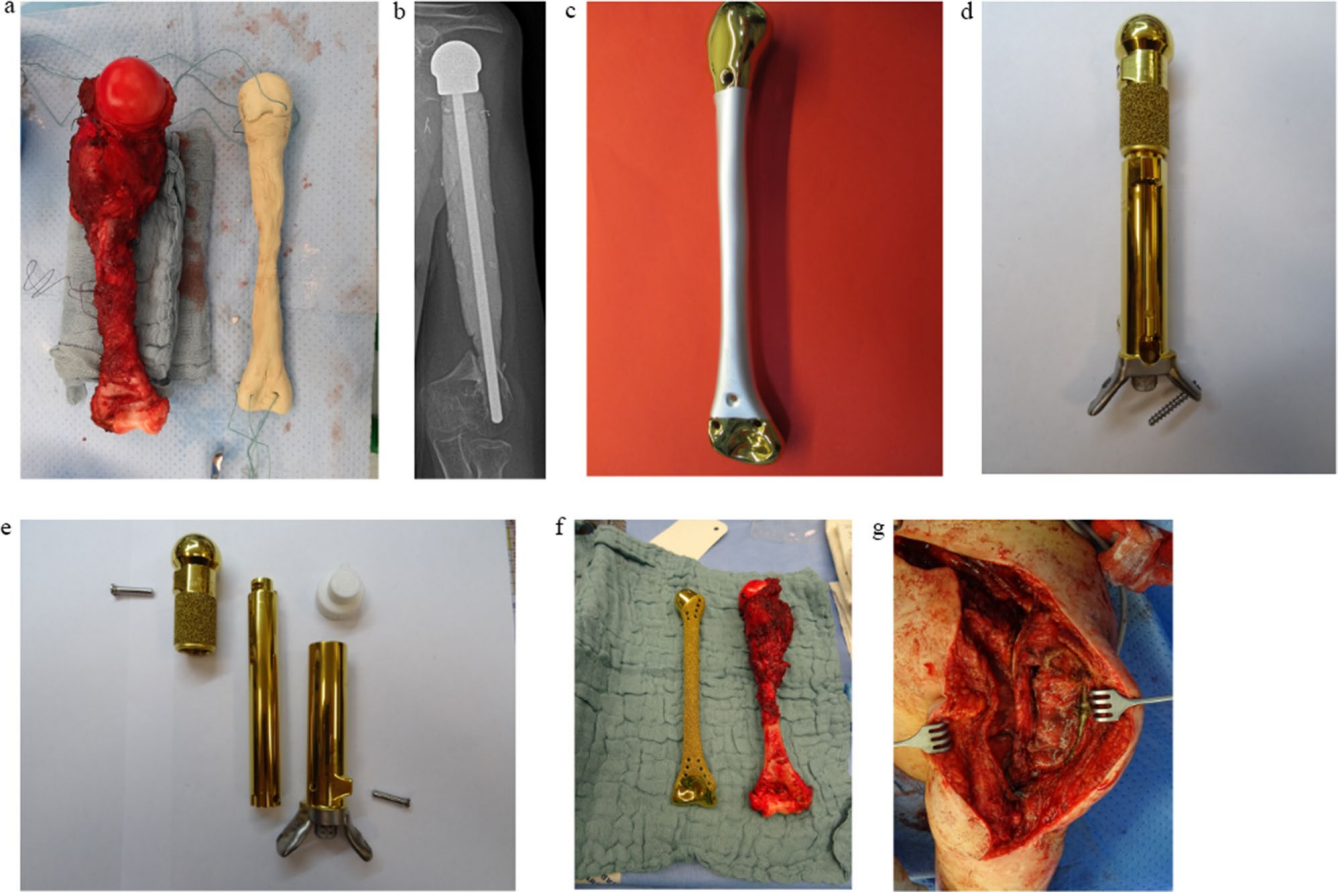
Overview over different reconstruction types ***a***. Resection specimen of a total humerus and PMMA spacer modelled ex situ with proximal and distal suture augmentation. ***b*** Postoperative radiograph of a patient reconstructed with a PMMA spacer (using a 28 mm femoral head) and central rod lateralization (patient #1). ***c*** Polished total humerus reconstruction with silver coating on the implant body (patient #3). ***d*** Assembled Xpand^®^ proximal humerus reconstruction with a distal custom-made hollow stem with additional plates (patient #1). ***e*** Disassembled Xpand^®^ proximal humerus reconstruction (patient #1). ***f*** Resection specimen of a total humerus and an EPORE^®^ total humerus (patient #6). ***g*** Transposed clavicle after CPH procedure (patient #4)

After total humerus resection, PMMA spacers are modeled ex situ during the operation using the resected bone’s dimensions and proportions as a template. They are implanted after the hardening process has concluded. The articulating joint surfaces are augmented using sutures embedded into the hardening bone cement, to be used as suture osteosyntheses and a means to prevent instability and dislocation (Fig. 2a).

After subtotal humerus resection, PMMA spacers are molded around a central titanium or steel rod, which is fixed into the remaining intramedullary canal of the distal humerus. Suture osteosyntheses are used at the proximal glenohumeral joint surface to prevent instability and dislocation *(*Fig. 2b).

#### Polished total humerus reconstruction (*n* = 2)

First-generation custom-made 3D printed monobloc proximal and distal hemiprosthesis with a smooth surface, optional silver coating and joint-sided suture holes to prevent implant instability and dislocation (Figs. 2c).

#### Xpand^®^ total humerus (*n* = 2) and Xpand^®^ proximal humerus with a custom-made hollow distal humerus stem and additional plates (*n* = 1)

Custom-made growth endoprosthesis with a motor and receiver unit for transcutaneous elongation of the upper arm (5 cm) (Fig. 2d-e) in patients with a(n) (expected) secondary limb length discrepancy and sufficient viable soft tissue coverage of the implant.

#### EPORE^®^ total humerus (*n* = 2)

Second-generation custom-made 3D printed monobloc proximal and distal hemiprosthesis with a highly cancellous implant body surface and a joint-sided polished surface with adjacent suture holes to prevent implant instability and dislocation (Fig. 2f).

#### Clavicle pro humero (*n* = 1)

A reconstruction technique reported by Winkelmann in 1992 [3] (Fig. 2g).

The clavicle is mobilized by arthrotomy of the sterno-clavicular joint. After transposition by approximately 90° in the acromio-clavicular joint, it is fixed to the remaining humerus.

### Complications and complication management in follow-up

Follow-up recommendations included quarterly presentations to the outpatient clinic within the first two years, biannual presentations until completion of the fifth and annual presentation until completion of the tenth year after the operation. Presentations included patient history, a clinical examination and radiographs in two planes of the reconstruction. Functional outcome, the incidence, management and outcome of complications, which were classified according to Henderson [19], were documented in the hospital information system.

### Functional outcome

The Musculo-Skeletal Tumor Society (MSTS) score and Toronto Extremity Salvage Score (TESS) were used to assess functional outcome [20–21]. The respective questionnaires were handed out in paper form and completed as part of the outpatient follow-up appointments.

### Statistical analysis

The authors performed descriptive statistical analysis, calculating the frequency of occurrence and percentage of independent variables.

## Results

Limb salvage with an intact motor function of the lower arm, hand and fingers was achieved in 100% of cases. The mean follow-up in this patient collective is 89.8 months (range 27–123 months). Four patients are continuously disease-free at a mean follow up of 105.5 months (range 87–123 months). One patient is alive with disease at 27 months after the operation. Two patients subsequently died of their disease 13 and 34 months after primary tumor resection.

### Complications and complication management

The reconstruction survival (retention of the primary implant at the time of last follow-up) in this patient cohort was 28.6% (*n* = 2/7). However, two patients underwent a voluntary secondary procedure to address a limb length discrepancy by exchanging the primary reconstruction for a growing prosthesis. Therefore, the corrected reconstruction survival, if these patients are excluded, was 40% (*n* = 2/5). Apart from these two voluntary revision operations, seven revision operations were mandatory in order to address complications. Of thirteen observed complications, eight necessitated an operative revision (61.5%; *n* = 8/13). The observed complications will be detailed in the following paragraphs by order of decreasing frequency.

### Type 1a failure– Soft tissue failure– instability (*n* = 6)

#### Distal instability and dislocation (type 1a failure) (*n* = 5)

**Fig. 3:**
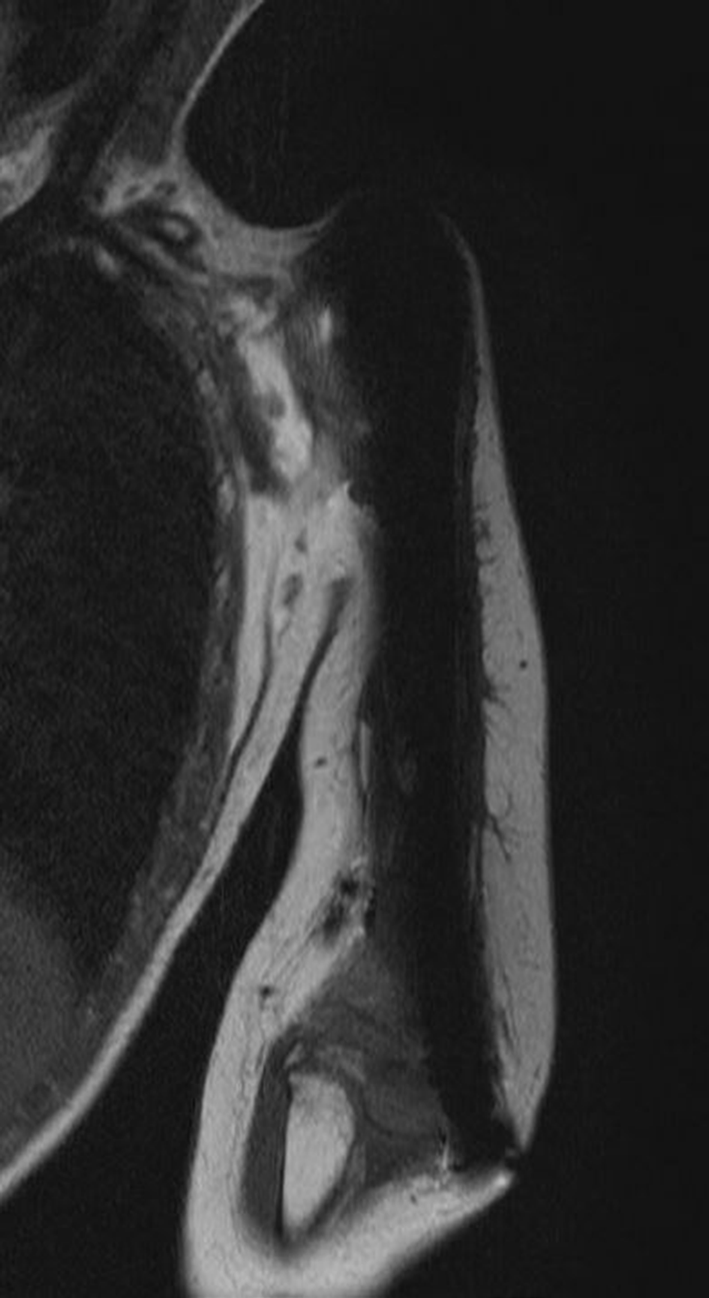
Coronar MRI image of a dislocated spacer with distal dislocation after total humerus resection and scarce soft tissue coverage (patient #4)

Distal instability and dislocation of the reconstruction were observed 5 times in two patients (#3 and 4; Fig. 3). Both patients, diagnosed with Ewing sarcoma and aged 4 years at the time of primary reconstruction, underwent total humerus resection.

Patient #3 underwent primary reconstruction using a PMMA spacer. Due to distal instability and dislocation of the spacer in the elbow joint, a revision operation and conversion to a custom-made polished total humerus reconstruction was performed 14 months after the primary operation. The instability persisted, leading to a pressure ulcer and concomitant periprosthetic infection of the implant within a month of implantation. To treat the infection, a two-stage approach, implanting another PMMA spacer, loaded with antibiotics, was performed. Despite a local muscle flap of the triceps muscle, to increase stability, and temporary immobilization of the elbow joint (which was performed after all operations), the instability persisted. A conservative attempt of closed reduction and putting on a plaster cast for six weeks (7 months after the 3rd operation), improved stability of the elbow joint only temporarily. Ultimately, the patient underwent a fifth revision operation. He was implanted with a growing prosthesis (Xpand^®^ total humerus) with a fixed-hinge joint mechanism and ulna stem.

Patient #4 suffered from distal instability after polished total humerus reconstruction (1st operation). After completion of her oncological treatment, including adjuvant chemotherapy and local radiation therapy of the humerus, she presented to the outpatient clinic with a distal instability and dislocation, threatening to breach the subcutaneous tissue and skin. She was revised 12 months after the primary operation. Reduction of the dislocation and a local muscle flap of the triceps muscle, to increase stability, were planned. During the procedure, the operating surgeon suspected bacterial contamination of the reconstruction (which was later disproven; normal white blood cell count and C - reactive protein levels before the operation, negative microbiological results in multiple specimen) and removed the prosthesis, implanting an antibiotic-loaded PMMA spacer instead. Another 16 months later, the patient presented with a recurrent distal dislocation. By that time, the elbow joint was rigid in an extended and dislocated position. The motor control of the elbow joint, remaining muscle coverage and tissue quality following radiation therapy were poor. As a result, the patient underwent a CPH reconstruction to improve mobility in the elbow joint, while accepting a significant shortening of the upper arm.

#### Proximal instability and dislocation (type 1a failure) (*n* = 1)

Patient #1 developed a proximal instability of the glenohumeral joint following the lengthening procedure of the upper arm using an Xpand^®^ prosthesis. Albeit asymptomatic, the patient is able to provoke a proximal and dorsal dislocation of her hemiprosthetic implant by flexing the biceps muscle. When relaxing the biceps muscle, the hemiprosthetic implant spontaneously resets.

### Periprosthetic infection (type 4 failure) (*n* = 2)

Periprosthetic infection occurred in two patients. Patient #2 developed localized hyperthermia, flush of the affected arm, and mildly elevated C - reactive protein (CRP) levels 56 months after his primary operation. He underwent a single stage revision with implantation of a new antibiotic releasing PMMA spacer and a 6-week antibiotic regimen for a low-grade infection of the reconstruction. Identification of a microbiological organism causing the infection failed (4/4 samples). So far, the patient showed no signs of persistent or recurrent periprosthetic infection in his follow-up since then.

Patient #3 developed a periprosthetic infection of his 2st reconstruction due to a pressure ulcer of the soft tissues caused by distal instability and dislocation of the elbow joint. Management of both instability and infection using a 2-stage approach was already presented in an earlier paragraph (see distal instability).

### Prosthetic failure (type 3a failure) (*n* = 2)

Prosthetic failure was observed in patient #1 who presented with distal rod migration of the PMMA spacer after subtotal humerus resection (Fig. 1b). The implant migration was asymptomatic and did not necessitate a revision operation. Yet, it was addressed in a 2nd operation, when an Xpand prosthesis was implanted to compensate for limb length discrepancy. Patient #2 presented with a central rod fracture of the PMMA spacer after subtotal humerus resection and adjuvant radiation therapy. In the absence of symptoms, the fracture did not lead to a revision operation. However, he ultimately underwent a second operation for a low-grade infection of the reconstruction (see periprosthetic infection).

### Aseptic loosening (type 2a failure) (*n* = 1)

Patient #3 presented with temporary radiographic signs of incomplete ingrowth of the ulna stem of his Xpand^®^ total humerus prosthesis. However, he was asymptomatic and ingrowth occurred spontaneously over time without an additional intervention.

### Local recurrence (type 5 failure) (*n* = 1)

Patient #6, who was diagnosed with a V2/R0 status after resection of his high-grade osteosarcoma, developed a local soft tissue recurrence 25 months after tumor resection. Local recurrence was associated with a recurrence of multiple pulmonary metastases. Therefore, the patient currently receives palliative chemotherapy. An operation of his asymptomatic local recurrence is not planned due to the palliative treatment intent.

### Biological reconstruction failure (*n* = 1)

**Fig. 4:**
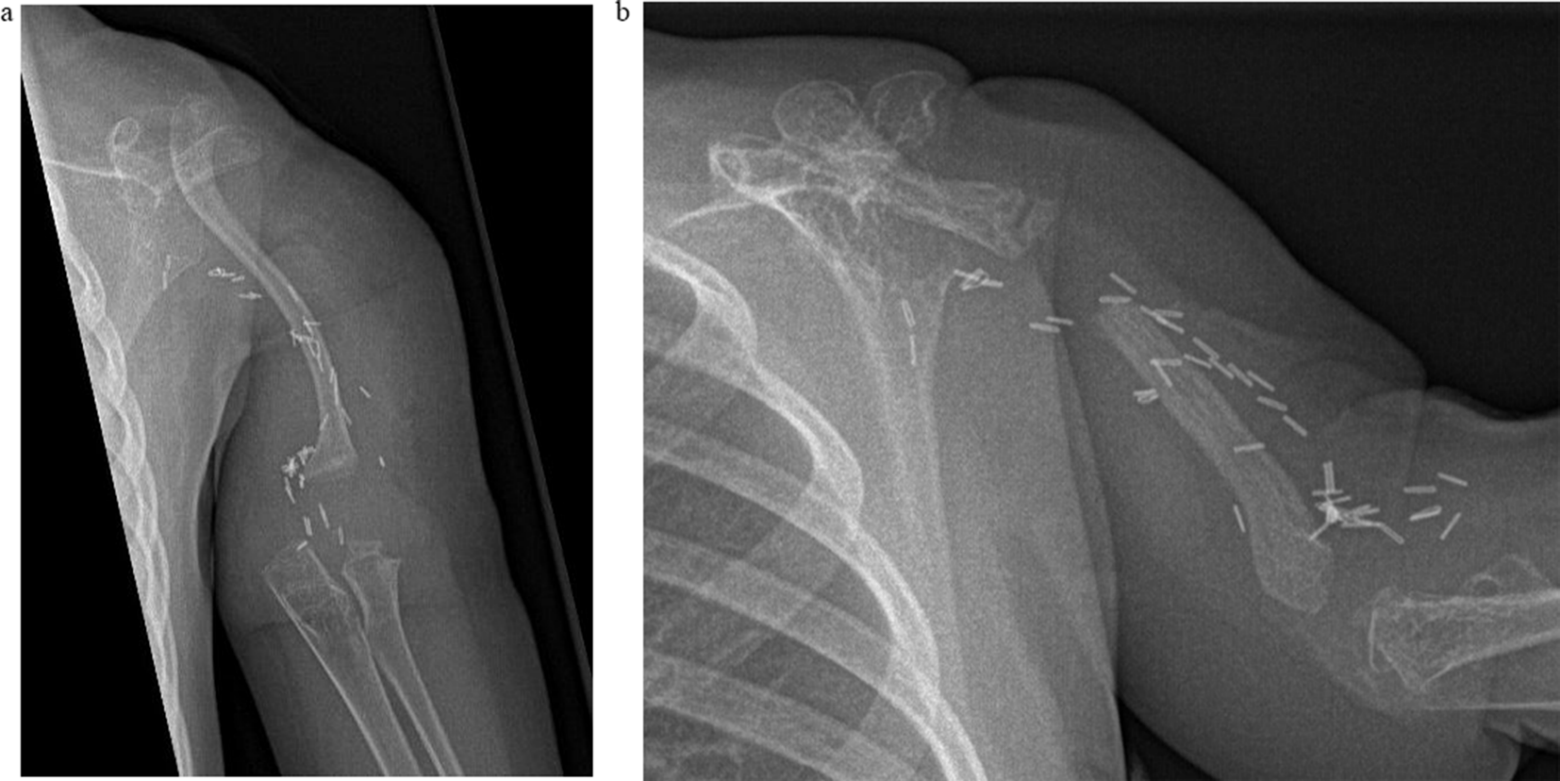
Postoperative radiographs after CPH reconstruction. Fracture and osteolysis were incidentally observed 12 months after the reconstruction (**a**). Constant presentation of a central osteolysis of the clavicle while retaining arm length and stability in both joints (**b**) (patient#4)

Patient #4 ultimately received a CPH reconstruction (Fig. 4). The postoperative radiographs showed an asymptomatic fracture and osteolytic pseudarthrosis of the clavicle 12 months after the operation. However, upper arm length remains stable without proximal or distal instability of the glenohumeral or elbow joint.

An overview of revision operations and complications is depicted in Table 2.

### Impact of adjuvant radiotherapy (*n* = 4)

Adjuvant radiotherapy was performed in three patients with a Ewing sarcoma and one patient with an osteosarcoma. In both Ewing sarcoma patients, who received radiotherapy due to a poor response of the tumor to chemotherapy (#2 and 4), and one Ewing sarcoma patient with a V1 status in the histopathological analysis (#7), we did not observe a local recurrence until last follow-up (*n* = 2) or death of disease (*n* = 1). The only local recurrence, associated with pulmonary metastases, was observed in the osteosarcoma patient (#6, V2).

Adjuvant radiation therapy did not lead to local wound complications; however, the soft tissue quality in patients #2 and 4 suffered significantly, leading to stiffened and fibrotic muscle tissue and ultimately limiting secondary reconstructive options. In addition, radiation therapy may have contributed to the low-grade implant-associated infection, observed in patient #2.

### Functional outcome

#### Lengthening of growing prostheses (Xpand^®^ subtotal and total humerus (*n* = 1 and *n* = 2)

**Fig. 5:**
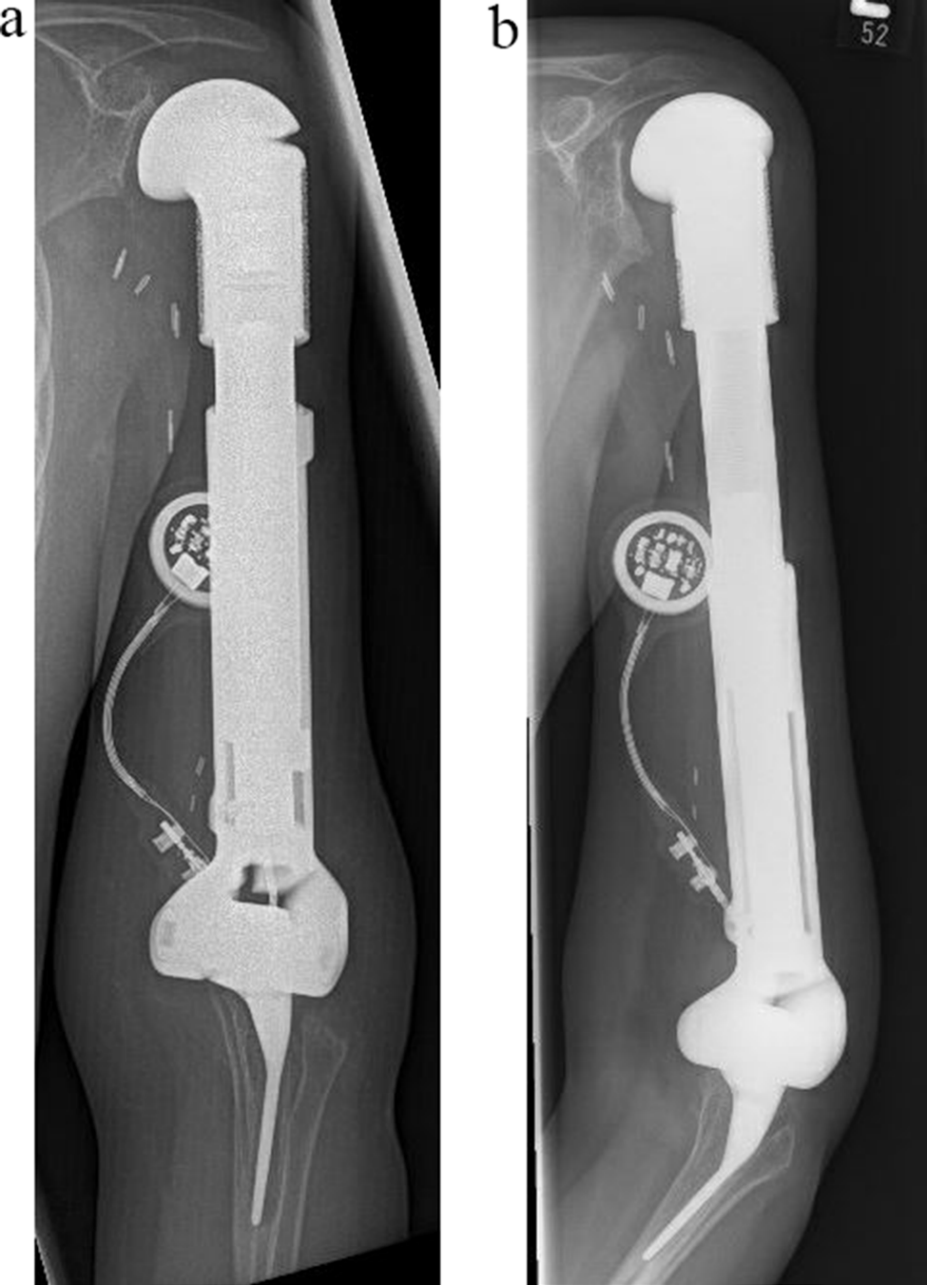
Postoperative radiographs at the beginning (**a**) and after completion (**b**) of the elongation process of the Xpand^®^ growing prosthesis (patient #3)

The growing prostheses used in this patient cohort, allowed an extension of the implant of 5 cm. Two patients were able to elongate their implant by the full 5 cm (Fig. 5). Since the upper extremity is exposed to a decreased load, when compared to the lower extremity, patients continued their daily lives with the extended growing prosthesis without additional operations. Lengthening-associated complications, as observed in the lower extremity, such as motor damage with incomplete extension, breakage of the cable between motor and subcutaneous receiver or loss of already elongated distance, did not occur in this cohort. One patient died of disease before the maximum extension of the implant was reached.

#### Reconstruction stability after using EPORE^®^ coating on subtotal and total humerus reconstructions

**Fig. 6:**
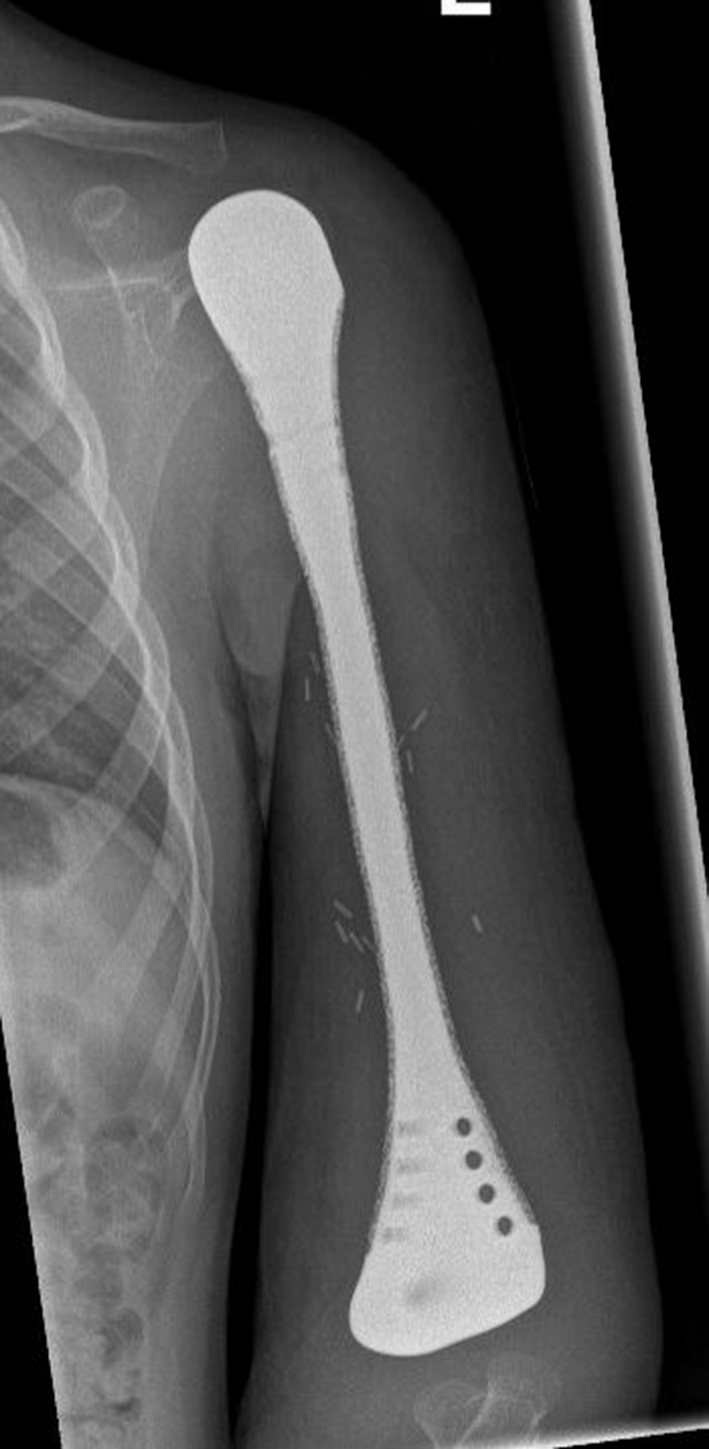
Postoperative radiograph of a total humerus reconstruction with an EPORE^®^ coating (patient #7)

The use of partial or total EPORE^®^ coating of the implants (except for joint surfaces) led to stable joints in five patients (patients #1,3,5–7). In patients 1, 3 and 5 an EPORE ^®^ ring was added to the proximal design of the Xpand^®^ prosthesis (Figs. 2d-e and 5). In patients 6 and 7, the total humerus implant bodies were covered with an EPORE^®^ coating, leading to stable reconstructions in both cases (Fig. 6).

#### Handedness

As the majority of patients in this cohort were operated before starting primary school and learning how to write, they developed a handedness, which is contralateral to their operated arm.

#### ROM, MSTS and TESS scores

After subtotal resection of the humerus and salvage of the elbow joint, patient #1 and #2 achieve an extension/flexion of 0/20/120° and 0/40/120° in the elbow joint. Both patients are able to touch their mouths with their hand. Pro- and supination are not limited significantly.

Patients #3 an #5, reconstructed with a fixed-hinge total humerus growing prosthesis, achieve(d) an extension/flexion of 0/30/95° and 0/40/90° (patient #5 died 12 months after reconstruction). Pro- and supination are/were approximately half of their normal range.

Patients #6 and #7, reconstructed with an EPORE^®^ total humerus hemiprosthesis, achieve(d) an extension/flexion of 0/30/100° and 0/10/130°. Pro- and supination are/were limited by less than half of their normal range. Patient #6 was able to touch his mouth, perform full-weight push-ups and climb recreationally using both arms.

Patient #4, reconstructed with a CPH, has a very limited and poor active ROM of both the glenohumeral and elbow joint. However, she is able to perform complex tasks, such as braiding her hair, by passively swinging her hand (or guiding her arm using her healthy arm) and holding on to something (e.g. clothing) when her movement reaches its destination.

The mean MSTS score and TESS score in this patient cohort were 16.6 (range 7–26) and 51.5 (range 17.5–84).

Patients (*n* = 5) scored a mean of.

- 3.2 (modest; non-disabling; range 2–5) in the category “Pain”,
- 2 (> than a recreational restriction; range 0–5) in the category “Function”,
- 2.4 (satisfied; range 0–5) in the category “Emotional”,
- 3 (not above shoulder; limited pro-/supination; range 0–5) in the category “Hand positioning”,
- 3.2 (loss of fine movements; range 2–5) in the category “Manual dexterity” and.
- 2.8 (limited; range 0–4) in the category “Lifting ability”.

## Discussion

Despite extensive tumors leading to (sub-) total humerus resections in this pediatric patient cohort aged less than 10 years at the time of tumor resection and first reconstruction, limb salvage with an intact motor function of the lower arm, hand and fingers was achieved in 100% of patients (*n* = 7).

However, the absence of adequately dimensioned off-the-shelf endoprostheses or biological options bridging the extensive resection defects have taken their toll on reconstruction survival, which was only 28.6% (*n* = 2/7). This outcome was premeditated and a result of the high rate of primary reconstructions using a PMMA spacer (*n* = 4), as time to the scheduled operation was frequently too short to plan and manufacture a custom-made implant. Nonetheless, when excluding patients, who chose to undergo revision to reduce arm length discrepancies in the absence of other complications, reconstruction survival still remained low at only 40% (*n* = 2/5).

In this patient cohort, 61.5% of complications (*n* = 8/13) necessitated additional surgeries (*n* = 7).

Distal instability was the most frequently observed complication (*n* = 5) in this cohort, highlighting the importance of salvaging even short segments of remaining bone of the distal humerus, whenever oncologically feasible. In this case series, polished total humerus implants, as well as PMMA spacers, were prone to instability and dislocation, which led to disuse of polished silver-coated total humerus implants in the course of the observation period. In literature, only proximal instability and dislocation are described. However, Tsuda et al. report a higher risk of proximal subluxation in a patient population aged less than 9 years in their study about the long-term outcomes of extendable endoprostheses of the (proximal) humerus in children (*n* = 35; median age 9 years) [17]. Meanwhile, the design of a total humerus prosthesis with a highly cancellous implant body was not susceptible to instability or dislocation. As other studies have reported, highly cancellous implant surfaces allow ingrowth of reactive soft tissue into the highly cancellous implant surface [22–23], which is likely to be the main reason for an increased implant stability. On the downside, said ingrowth tends to rule out secondary arm lengthening, as conversion of the EPORE^®^ implant to a growing prosthesis carries a higher risk of neurovascular damage.

The two cases of periprosthetic infection in this study were associated with concomitant dislocation and skin breaches or adjuvant radiation therapy in one case each. This is in accordance with other published data, which supports that soft tissue failure and periprosthetic infection have a negative impact on reconstruction survival [24].

Central rod fracture and migration of PMMA spacers (each *n* = 1) were comparatively minor complications in this series. Due to a lack of load on the upper extremity, when compared with the lower limbs, and a restricted range of motion of the glenohumeral joint, both observed complications did not lead to additional operations. For this reason, the use of PMMA spacers, which are only used as temporary reconstructions in the lower limb, are an accepted– possibly even permanent– reconstruction technique in the upper extremity [25].

Compared with the lower extremity, reports on the outcome of growing prostheses and lengthening of the upper extremity are less frequent. However, there are studies by Ayoub et al. and Beebe et al. reporting on successful lengthenings in small patient collectives [11–12]. In this study, we report two successful cases of arm lengthening in patients who did not receive adjuvant radiation therapy. Compared to a significant functional enhancement of the lower extremity in terms of gait and walking disability, it appears to have mostly cosmetic implications in the upper extremity. For this reason, the authors believe that arm lengthening should only be considered in patients with a low risk profile (non-irradiated, sufficient soft tissue coverage of the implant) and an expressed preference to undergo this procedure.

Despite retaining a useful hand and elbow joint, functional limitations after (sub-) total humerus reconstructions become apparent as all but one patient underwent reconstruction before school enrollment and subsequently trained their healthy upper limb as their dominant side. This is supported by the results of the MSTS and TESS questionnaires, which reveal that patients report limitations in range of motion (usually ROM above the waist but not above the shoulder), fine movements and strength. Both MSTS and TESS scores in this study are lower than results reported by others. For instance, Tsuda et al. observed MSTS scores ranging between 73 and 90% in their collective of 35 patients treated with an extendable endoprosthetic replacement of the proximal humerus [17]. Even so, the comparability of this and other studies with our findings is limited due to different inclusion criteria (i.e. shorter reconstructions, different age distribution within patient collective, inclusion of various tumor sites, one affected joint vs. two joints affected by total humerus resection).

## Conclusions

Limb salvage, using custom-made endoprosthetic prostheses, is a feasible treatment option for extensive bone defects of the humerus after (sub-) total resections despite young patient age. Nevertheless, even short segments of the distal humerus should be salvaged, whenever oncologically feasible, to prevent distal instability. While a PMMA spacer is a reasonable temporary or permanent reconstruction option, definitive reconstructions, such as (growing) prostheses or prostheses with a highly cancellous implant body surface, show the highest promise in terms of arm elongation, joint stability and permanence in this study. However, growing prostheses should only be considered in patients who have sufficiently viable non-irradiated soft tissue coverage of the implant and express a strong preference for lengthening of their arm, as it is mostly cosmetic with only little impact on functional considerations. Custom-made prostheses with highly cancellous implant body surfaces should be considered as a permanent reconstruction whenever the amount of viable soft tissue coverage is more limited and/or in the event of necessary additional radiotherapy. It is important to counsel patients with regard to the probable limitations of the affected limb in terms of range of motion, fine movements of the hands and fingers and strength despite an intact innervation.

### Limitations

This study represents the authors’ interpretation of the outcomes, complications and complication management after (sub-) total humerus resection in skeletally immature patients (*≤* 10 years). The treatment recommendations are provisional and may evolve in the course of larger follow-up studies. We acknowledge the shortcomings of a retrospective study design. The small number of included patients limits the significance of our findings and further studies are necessary to support and/or extend our knowledge about the outcomes of this subgroup of patients.

## Data Availability

No datasets were generated or analysed during the current study.

## Abbreviations

ROM: Range Of Motion
MSTS: Musculo-Skeletal Tumor Society
TESS: Toronto Extremity Salvage Score
VFET: Vascular Fibular Epiphyseal Transfer
PMMA: PolyMehtyl MethAcrylate
EPORE®: Proper name for implant with a highly cancellous implant surface
3D: Three-Dimensional
CT: Computed Tomography
DICOM: Digital Image and Communications in Medicine standard (data format)
CAD: Computer Aided Design
MRI: Magnetic Resonance Imaging
